# Interrupted time series analysis of the impact of the COVID-19 pandemic and compulsory social health insurance system on fertility rates: a study of live births in Kazakhstan, 2019–2023

**DOI:** 10.3389/fpubh.2024.1454420

**Published:** 2024-08-23

**Authors:** Indira Karibayeva, Sharapat Moiynbayeva, Valikhan Akhmetov, Sandugash Yerkenova, Kuralay Shaikova, Gaukhar Moshkalova, Dina Mussayeva, Bibinur Tarakova

**Affiliations:** ^1^Department of Health Policy and Community Health, Jiann-Ping Hsu College of Public Health, Georgia Southern University, Statesboro, GA, United States; ^2^Department of Science and Consulting, Kazakhstan Medical University “KSPH”, Almaty, Kazakhstan; ^3^Department of Economics of Healthcare and Insurance Medicine, Kazakhstan Medical University “KSPH”, Almaty, Kazakhstan; ^4^Department of Epidemiology, Evidence-Based Medicine and Biostatistics, Kazakhstan Medical University “KSPH”, Almaty, Kazakhstan; ^5^Department of Pediatrics, Siberian State Medical University, Tomsk, Russia; ^6^Department of Visual Diagnostics, Asfendiyarov Kazakh National Medical University, Almaty, Kazakhstan

**Keywords:** COVID-19, fertility rates, live birth, maternal and child health, Kazakhstan

## Abstract

**Introduction:**

The COVID-19 pandemic triggered global health crises, affecting population health directly through infections and fatalities, and indirectly by increasing the burden of chronic diseases due to disrupted healthcare access and altered lifestyle behaviors. Amidst these challenges, concerns regarding reproductive health and fertility rates have emerged, necessitating an understanding of their implications for policymaking and healthcare planning. Furthermore, Kazakhstan’s healthcare landscape underwent significant changes with the reintroduction of compulsory social health insurance system in January 2020, coinciding with the onset of the COVID-19 pandemic. This study aims to assess the impact of the COVID-19 pandemic and compulsory social health insurance system on fertility rates in Kazakhstan by examining live birth data from 2019 to 2024.

**Methods:**

Using Interrupted Time Series analysis, we evaluated the effect of the COVID-19 lockdown announcement and compulsory social health insurance system implementation on monthly birth rates, adjusted for the number of women of reproductive age from January 2019 to December 2023.

**Results:**

In the final model, the coefficients were as follows: the effect of the COVID-19 lockdown was estimated at 469 (SE = 2600, *p* = 0.8576); the centering variable was estimated at 318 (SE = 222, *p* = 0.1573), suggesting no significant trend in monthly birth rates over time; the insurance effect was estimated at 7,050 (SE = 2,530, *p* < 0.01); and the effect of the number of women of reproductive age was estimated at -0.204 (SE = 0.0831, *p* = 0.01).

**Discussion:**

The implementation of the compulsory social health insurance system, rather than the announcement of the COVID-19 lockdown, has had a significant positive impact on live birth rates in Kazakhstan. However, despite governmental efforts, live birth rates are declining, potentially due to unaddressed health needs of fertile women and economic challenges. Urgent policy-level actions are needed to address gaps in healthcare services and promote reproductive health.

## 1 Introduction

Live birth rates are a critical indicator of population health and demographic trends (1), reflecting a society’s reproductive behaviors in response to local and global economic and political situations. Globally, birth rates are declining, and Kazakhstan is no exception (2). Historical data indicates that the live birth rate is highly sensitive to the nation’s well-being. Following the collapse of the Soviet Union, Kazakhstan experienced a significant drop in live birth rates, with the lowest in 1999, reflecting the socio-economic challenges of transitioning to an independent state and facing substantial population migration (3, 4). However, since 2003, the population has grown, aided by the cessation of migration and an increase in birth rates (3). Despite this growth, the last decade has seen a steady decline in live birth rates in Kazakhstan, dropping from 22.620 per 1,000 people in 2013 to 17.477 per 1,000 people in 2023, according to Macrotrends data (5). Understanding these fluctuations is essential given Kazakhstan’s complex socio-economic landscape, shaped by recent healthcare reforms and global events.

The Coronavirus Disease 2019 (COVID-19) pandemic was a global health crisis characterized by the rapid spread of a coronavirus, causing widespread illness and mortality (6, 7). This unprecedented health emergency not only directly impacted population health through infections and fatalities but also indirectly contributed to an increased burden of chronic diseases due to disruptions in healthcare access, delayed treatments, and changes in lifestyle behaviors (8). Furthermore, the pandemic raised reproductive health concerns and affected fertility rates globally (9, 10). Understanding the pandemic’s impact on fertility patterns is crucial for informed policymaking and healthcare planning (11).

Kazakhstan, presents a unique case study due to its evolving healthcare landscape during the pandemic. As of October 2023, the Johns Hopkins Coronavirus Resource Center reported 1,498,668 confirmed COVID-19 cases and 19,071 deaths in Kazakhstan (12). It was estimated that at the peak of the outbreak, Kazakhstan would need approximately 47,248 beds for severe cases and 1,930 beds for critical cases (13). Additionally, it was projected there would be a significant demand for healthcare workers and both diagnostic and treatment equipment (13). The pandemic exposed vulnerabilities in the healthcare system, particularly the severe shortages of essential medications and equipment, as well as the insufficient number of hospital beds (14).

In January 2020, the reintroduction of the compulsory social health insurance system (CSHIS) commenced, coinciding with the onset of the COVID-19 pandemic in March 2020 (15–17). Prior to this, Kazakhstan’s healthcare was predominantly state-funded under universal healthcare coverage, offering limited publicly funded services and relying heavily on out-of-pocket payments due to underfunding (18). Inadequate state funding and inefficient strictly centralized management structure led to poor quality and inaccessibility of medical services (19, 20). The CSHIS aimed to enhance healthcare quality and accessibility by reforming funding principles and increasing state healthcare financing (21). The system’s implementation resulted in a more than 2.5-fold increase in healthcare sector funding over three years, from approximately 1 trillion KZT in 2019 to over 2.5 trillion KZT in 2023 (22).

According to Kazakhstan’s public health and healthcare legislation, pregnant women, children, and women with children under one year of age were entitled to a guaranteed volume of free medical care under universal health coverage even before the introduction of the CSHIS in 2020 (23). However, limited healthcare funding led to high out-of-pocket costs, affecting accessibility and affordability of healthcare services (23). The WHO report on sexual, reproductive, maternal, newborn, child, and adolescent health showed that out-of-pocket spending accounted for 36% of total health expenditures in 2016, exceeding the WHO estimate of 20% or lower for adequate financial protection (18). By 2020, this out-of-pocket spending had decreased to 33.8% (21).

Abrokwah et al. have previously hypothesized that the introduction of social health insurance, analogous to a positive income shock, may influence fertility rates by reducing childbirth and pediatric healthcare expenses (24). After the rollout of the national health insurance program in Ghana, the authors found a pro-cyclical fertility effect at the individual level (24). However, no studies have examined the changes in live birth rates following the CSHIS implementation in Kazakhstan.

In this study, we aim to assess the impact of the COVID-19 pandemic and the compulsory social health insurance system implementation on fertility rates in Kazakhstan, focusing on live births from 2019 to 2023, using Interrupted Time Series (ITS) analysis.

ITS analysis is a statistical method used to evaluate the impact of population level interventions that have a specific start date on outcomes measured over time (25). ITS was chosen for this study because it can pinpoint the effect of an intervention at the time of implementation while accounting for overall trends and confounding factors. This approach is particularly valuable for understanding how interventions like the COVID-19 lockdown and the introduction of the CSHIS have influenced fertility patterns in Kazakhstan.

## 2 Materials and methods

### 2.1 Data sources

The data for live birth rates in absolute numbers in Kazakhstan were obtained from the official website of the Statistical Committee of the Republic of Kazakhstan (26). This dataset provides monthly live birth counts from January 2019 to December 2023. Monthly data was obtained and combined.

**Figure 1:**
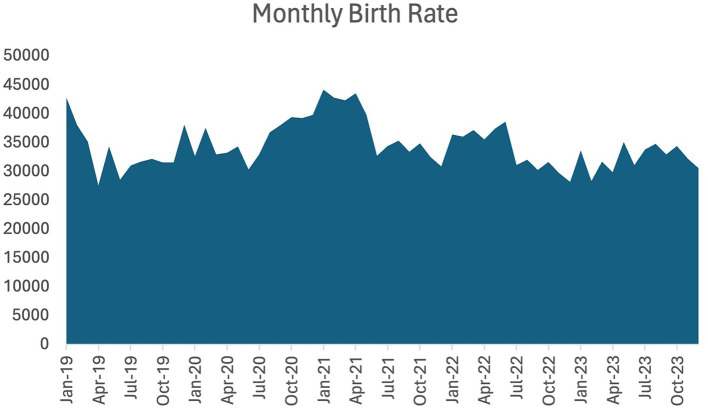
Monthly birth rate in Kazakhstan in absolute numbers (2019–2023).

The monthly birth rates, as reported by the Statistical Committee of the Republic of Kazakhstan, account only for infants for whom birth certificates were generated by the Civil Registry Office. Figure 1 presents the monthly live birth dynamics, illustrating an increase in the number of live births in 2020 and 2021, followed by decreases in 2022 and 2023.

**Figure 2:**
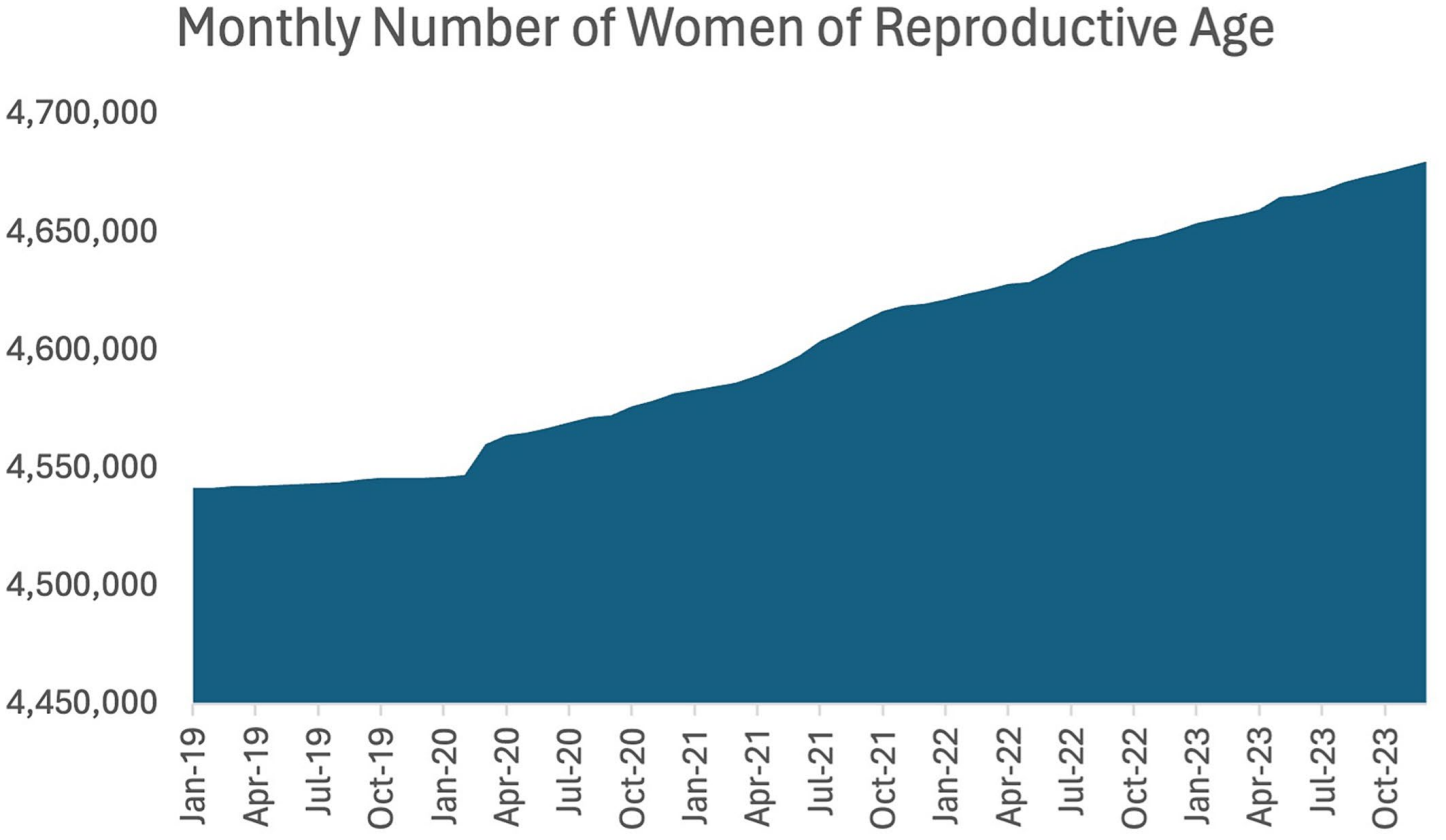
Monthly number of women of reproductive age (15–49 years).

The monthly number of women of reproductive age (15 to 49 years of age) for the years of 2019 to 2023 were also obtained from the Statistical Committee of the Republic of Kazakhstan website (27). Monthly data was obtained and combined. Figure 2 represents the monthly number of women of reproductive age.

### 2.2 Analysis method

We employed the Interrupted Time Series (ITS) analysis to assess the impact of the COVID-19 pandemic and CSHIS reintroduction on fertility rates in Kazakhstan. Interrupted time series analysis is a robust method for evaluating the effects of interventions or external events on time-series data. It allows for the examination of both immediate and gradual changes in the outcome variable following the intervention, in this case, the onset of the pandemic and the CSHIS reintroduction.

### 2.3 Data preprocessing

We established three variables, namely the “treatment” variable, the “centering” variable and the “CSHIS effect” variable, for the ITS analysis. The “treatment” variable represents the effect date, which was derived from the lockdown declaration date in Kazakhstan on March 19, 2020. This date was adjusted to account for a normal gestation period, resulting in the earliest potential response approximately nine months later, in December 2020. Therefore, the effect date corresponds to December 2020. The “centering” variable quantifies the temporal proximity of each monthly birth rate to the effect date. The “CSHIS effect” variable represents the effect date, which was derived from the CSHIS implementation date in Kazakhstan on January 1st, 2020. This date was adjusted to account for a normal gestation period, resulting in the earliest potential response approximately nine months later, in September 2020. Therefore, the effect date corresponds to September 2020. Consequently, we collected approximately two years of data preceding the anticipated impact of the COVID-19 lockdown. We then compared these trends with nearly three years of post-lockdown data.

### 2.4 Statistical analysis

All data processing and statistical analyses were performed using RStudio (Version 4.3.2; RStudio, Inc., Boston, MA, United States). Linear regression analysis was conducted using the ‘lm’ function. The unadjusted impact of the COVID-19 lockdown on birth rates in Kazakhstan was calculated based on the “treatment” and “centering” variables only. The impact of the COVID-19 lockdown on birth rates in Kazakhstan, adjusted for the number of fertile women, was calculated based on the “treatment,” “centering” variables, and the number of fertile women. The impact of the COVID-19 lockdown on birth rates in Kazakhstan, adjusted for the CSHIS effect, was calculated based on the “treatment,” “centering” variables, and the “CSHIS effect.” The final model included all variables. The ‘predict’ function, in conjunction with the ‘lm’ function, was employed to estimate live birth rates based on the date of COVID-19 lockdown implementation. The ‘plot’ function was used to create a scatter plot. Notably, all these functions are built into base R and do not require additional packages.

## 3 Results

### 3.1 Unadjusted impact of the COVID-19 lockdown on birthrates in Kazakhstan (2019–2023)

The ITS analysis examined the impact of the COVID-19 lockdown announcement date on monthly birth rates in Kazakhstan from January 2019 to December 2023. The unadjusted regression model results are presented in Table 1.

**Table 1:** Unadjusted linear regression model results examining the impact of the COVID-19 lockdown on birthrates in Kazakhstan (2019–2023).

Variables	Estimate	SE*	*p*-value
MBR**	32115.92	984.95	<0.001
Lockdown effect	5736.57	1820.47	<0.01
Centering trend	−183.84	51.11	<0.001

*SE—standard error; **MBR—monthly birth rate.

The intercept, representing the baseline monthly birth rate when other variables are held constant, was estimated at 32,115.92 (SE = 984.95, *p* < 0.001). The coefficient for the COVID-19 lockdown effect, indicating the change in the monthly birth rate nine months after the lockdown announcement, was estimated at 5,736.57 (SE = 1,820.47, *p* < 0.01). This suggests a significant increase in monthly birth rates nine months following the COVID-19 lockdown announcement. Additionally, the coefficient for the centering variable was estimated at −183.84 (SE = 51.11, *p* = 0.001), indicating a significant decreasing trend in monthly birth rates over time.

**Figure 3:**
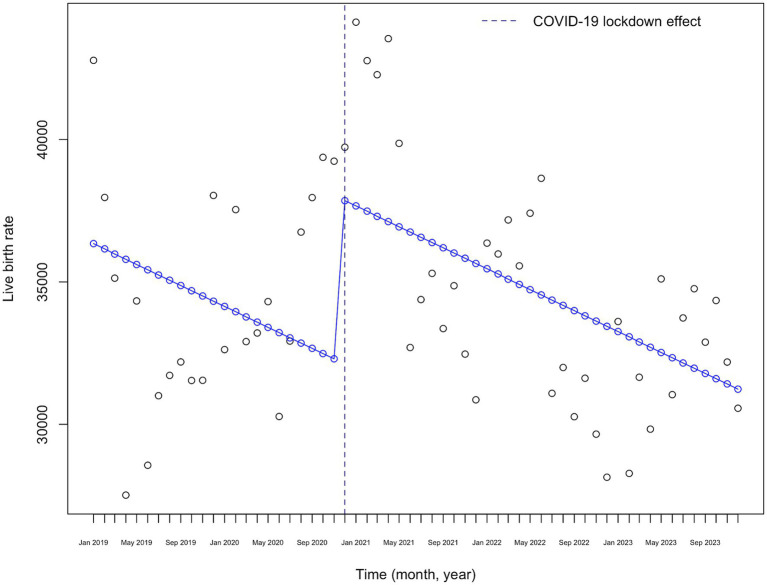
COVID-19 lockdown effect on monthly live birth rates in Kazakhstan (2019–2023).

Figure 3 presents the visual representation of the interrupted time series (ITS) analysis of monthly birth rates from 2019 to 2023. A significant increase in monthly birth rates is observed precisely at the “lockdown effect” point, which occurs nine months after the lockdown announcement. Following this peak, a downward trend in birth rates is evident.

### 3.2 The impact of the COVID-19 lockdown on birthrates in Kazakhstan adjusted to the number of fertile women

As a second step of the analysis, we adjusted the live birth rates based on the number of women of reproductive age in each month, as presented in Table 2. The intercept, representing the baseline monthly birth rate when other variables are held constant, was estimated at 1,120,000 (SE = 399,000, *p* < 0.01). The coefficient for the COVID-19 lockdown effect, representing the change in the monthly birth rate nine months after the COVID lockdown announcement, was estimated at 6,110 (SE = 1,730, *p* < 0.001). This indicates a significant increase in monthly birth rates nine months following the announcement of the COVID lockdown. Additionally, the coefficient for the centering variable was estimated at 432 (SE = 231, *p* = 0.06), indicating no significant trend in monthly birth rates over time. The coefficient for the number of women of reproductive age was estimated at −0.24 (SE = 0.09, *p* < 0.01), suggesting a downward trend in monthly live birth rates as the number of fertile women increases.

**Table 2:** Linear regression model results examining the impact of the COVID-19 lockdown on birthrates in Kazakhstan adjusted to the number of fertile women.

Variables	Estimate	SE*	*p*-value
MBR**	1,120,000	399,000	<0.01
Lockdown effect	6,110	1730	<0.001
Centering trend	432	231	0.06
Number of WRA***	−0.24	0.09	<0.01

*SE—standard error; **MBR—monthly birth rate; ***WRA—women of reproductive age.

**Figure 4:**
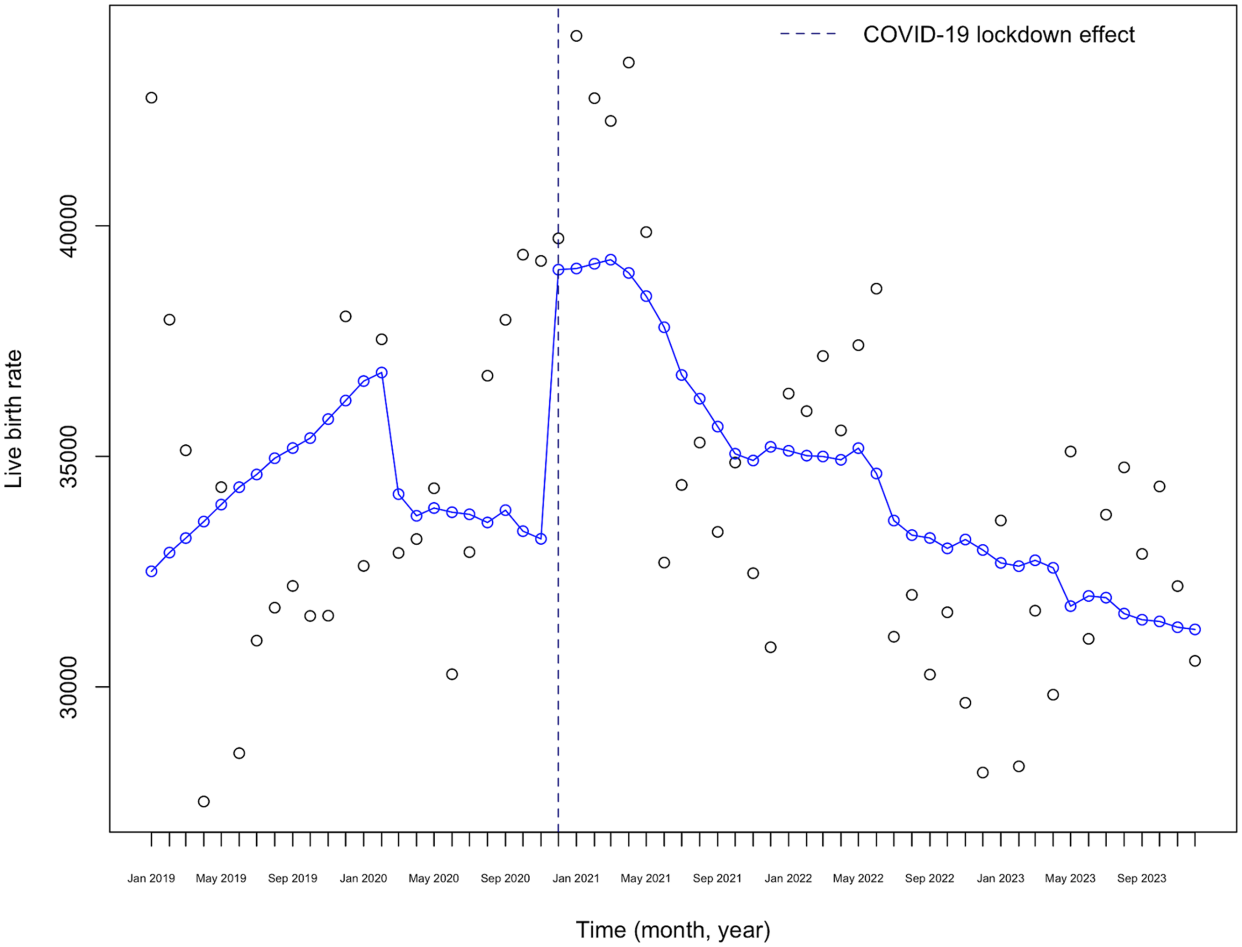
COVID-19 lockdown effect on monthly live birth rates adjusted to the number of fertile women in Kazakhstan (2019–2023).

Figure 4 shows the visual representation of the interrupted time series (ITS) analysis of monthly birth rates from 2019 to 2023, adjusted for the number of women of reproductive age. There is a marked decrease in the monthly birth rate starting in January 2020, followed by an increase at the time of the “lockdown effect” nine months after the lockdown announcement, with a fluctuating downward trend afterwards.

### 3.3 The impact of the COVID-19 lockdown on birthrates in Kazakhstan adjusted to the CSHIS effect

As the third step of the analysis, we adjusted the live birth rates for the “insurance effect” nine months after the announcement of the CSHIS, as presented in Table 3. The intercept, representing the baseline monthly birth rate when other variables are held constant, was estimated at 31,043 (SE = 986.7, *p* < 0.01). The coefficient for the COVID-19 lockdown effect, representing the change in monthly birth rates nine months after the COVID lockdown announcement, was estimated at −555.6 (SE = 2684.4, *p* = 0.8368). This indicates no significant change in monthly birth rates nine months following the COVID lockdown announcement. Additionally, the coefficient for the centering variable was estimated at −215.7 (SE = 48.9, *p* < 0.001), suggesting a significant downward trend in monthly birth rates over time. The coefficient for the insurance effect was estimated at 7,938.9 (SE = 2,618.8, *p* = 0.0037).

**Table 3:** Linear regression model results examining the impact of the COVID-19 lockdown on birthrates in Kazakhstan adjusted to the CSHIS effect.

Variables	Estimate	SE*	*p*-value
MBR**	31,043	986.7	<0.001
Lockdown effect	−555.6	2684.4	0.8368
Centering trend	−215.7	48.9	<0.001
CSHIS*** effect	7938.9	2618.8	0.0037

*SE—standard error; **MBR—monthly birth rate; ***CSHIS—Compulsory Social Health Insurance System.

**Figure 5:**
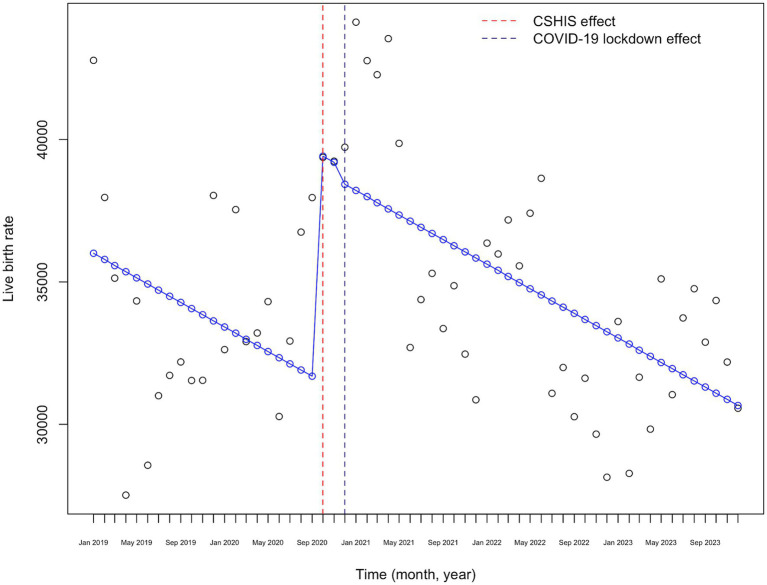
COVID-19 lockdown effect on monthly live birth rates adjusted for the CSHIS effect in Kazakhstan (2019–2023).

Figure 5 shows the visual representation of the ITS analysis of monthly birth rates from 2019 to 2023, adjusted for the CSHIS effect. There is a marked increase in monthly birth rates starting in September 2020, nine months after the implementation of CSHIS, followed by a slight decrease at the time of the “lockdown effect,” with a downward trend afterward.

### 3.4 The impact of the COVID-19 lockdown on birthrates in Kazakhstan adjusted to the number of fertile women and CSHIS effect

The final model shows monthly adjusted live birth rates considering the “CSHIS effect,” “COVID-19 lockdown effect” and the monthly number of fertile women, as presented in Table 4. The intercept, representing the baseline monthly birth rate when other variables are held constant, was estimated at 969,000 (SE = 381,000, *p* < 0.01). The coefficient for the COVID-19 lockdown effect, representing the change in monthly birth rates nine months after the COVID lockdown announcement, was estimated at 469 (SE = 2,600, *p* = 0.8576). This indicates a nonsignificant increase in monthly birth rates nine months following the COVID lockdown announcement. Additionally, the coefficient for the centering variable was estimated at 318 (SE = 222, *p* = 0.1573), suggesting no significant trend in monthly birth rates over time. The coefficient for the insurance effect was estimated at 7,050 (SE = 2,530, *p* < 0.01). The coefficient for the effect of the number of women of reproductive age was estimated at −0.204 (SE = 0.0831, *p* = 0.01).

**Table 4:** Adjusted linear regression model results examining the impact of the COVID-19 lockdown on birthrates in Kazakhstan.

Variables	Estimate	SE*	*p*-value
MBR**	969,000	381,000	<0.01
Lockdown effect	469	2,600	0.8576
Centering trend	318	222	0.1573
CSHIS*** effect	7,050	2,530	<0.01
Number of WRA	−0.20	0.08	0.01

*SE—standard error; **MBR—monthly birth rate; ***CSHIS—Compulsory Social Health Insurance System; ****WRA—women of reproductive age.

**Figure 6:**
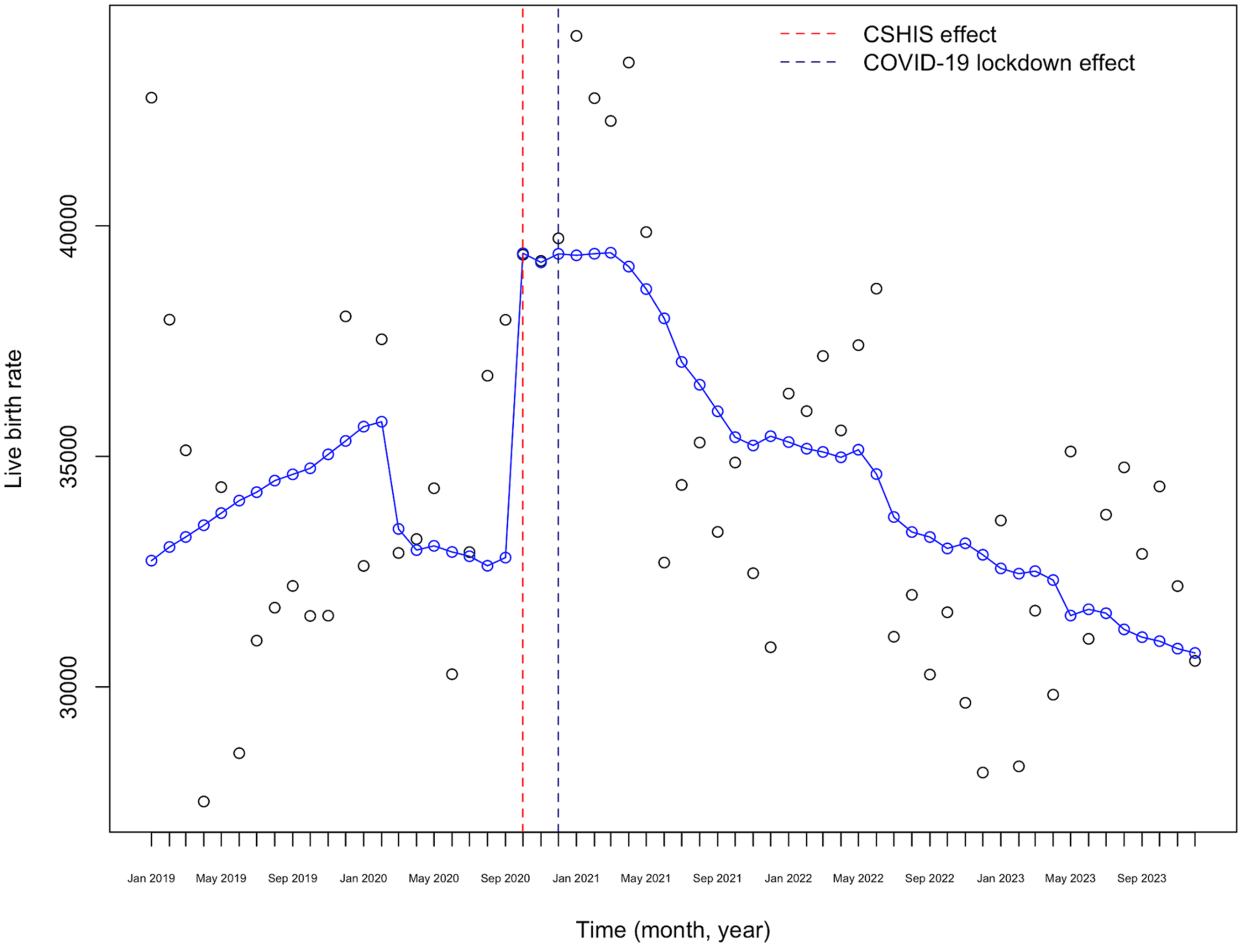
COVID-19 lockdown effect on monthly live birth rates adjusted for the CSHIS effect and the number of fertile women in Kazakhstan (2019–2023).

Figure 6 shows the visual representation of the ITS analysis of monthly birth rates from 2019 to 2023, adjusted for the CSHIS effect and the number of women of reproductive age. There is a marked decrease in monthly birth rates starting in January 2020, followed by an increase exactly nine months after the CSHIS implementation, with a fluctuating downward trend afterward.

## 4 Discussion

The COVID-19 pandemic has profoundly affected global public health, economies, and social dynamics. This study examines its specific impact on fertility rates in Kazakhstan using ITS analysis from 2019 to 2023, revealing significant fluctuations associated with pandemic events and the CSHIS implementation.

The unadjusted ITS analysis highlights a significant immediate effect of the COVID-19 lockdown on birth rates in Kazakhstan, with a notable increase nine months post-announcement. However, no significant long-term trends were detected, suggesting the effect was more immediate than sustained. In an analysis of the COVID-19 impact on birth rates in the United States, conducted by Stout and colleagues, they reported a 14% reduction in the initiation of pregnancy episodes following the societal shutdown due to the COVID-19 pandemic (28). This decline appeared to coincide with a reduction in conceptions following the mandated societal shutdown in March 2020. Similarly, a study conducted in Italy also indicated a decline in birth rates following the COVID-19 pandemic, particularly in three industrial cities (29).

The paramount effect of the COVID-19 lockdown, when analyzed further, was mitigated by the steadily increasing number of fertile women and the CSHIS implementation effect. The implementation of CSHIS in January 2020 significantly impacted fertility rates, leading to a notable increase in births nine months post-implementation. The initial phase of CSHIS introduction started in 2017, with full coverage effective from January 2020 (30). This policy aimed to enhance healthcare access and affordability, likely boosting reproductive confidence among couples. The significant coefficient for the CSHIS effect at 7,050 (SE = 2,530, *p* < 0.01) after adjusting for the “COVID-19 lockdown effect” and monthly number of fertile women underscores the importance of accessible healthcare services in influencing fertility decisions. CSHIS has the potential to provide continuous, stable funding to the healthcare sector, compared to the unsustainable fees paid by individuals for medical services (31). Additionally, CSHIS offers equitable access to healthcare services among populations with different income levels (31). Both factors play a substantial role in improving patient outcomes, which could explain the positive CSHIS effect on monthly birth rates in the country.

The economic fallout from the COVID-19 pandemic, characterized by job losses, income instability, and reduced economic growth, likely played a crucial role in the observed downward fertility trends following the introduction of the CSHIS. An analysis of the economic repercussions and policy implications of COVID-19 in Kazakhstan reveals that due to the substantial employment share of small-and medium-sized firms in the country, the short-term risk of job loss was enormous (32). Barrafrem et al. (33) conducted a study on people’s perceptions of the future economic situation within their households, the nation, and the world during the COVID-19 outbreak. They found that most individuals believed that their household economy would fare better than the national or global economy, which they explained through a “better-than-average effect.” The authors also highlighted the importance of improving perceived economic opportunities during national crises (33). Another study on Google Trends shows that during the outbreak, searches for boredom, loneliness, worry, and sadness increased, while searches for stress, suicide, and divorce decreased, indicating that the lockdown significantly impacted and shifted people’s mental health (34). In these scenarios, the role of governmental family-friendly policies aimed at supporting high birth rates in the country cannot be overestimated (35).

The “Ansagan Sabi” governmental program, which expanded access to *in vitro* fertilization procedures under the CSHIS, was launched in 2021 and exemplifies governmental family-friendly policies aimed at supporting birth rates in the country (36). However, our analysis reveals a concerning trend: despite adjustments made for the “lockdown effect,” “insurance effect,” and the growing number of women of reproductive age, live birth rates in Kazakhstan are declining. This downward trend, coupled with our recent health policy analysis identifying gaps in addressing the health needs of fertile women, particularly in predicting and preventing pregnancy-related cardiovascular complications such as gestational diabetes, gestational hypertension, and preeclampsia, underscores the urgent need for policy-level action to address this issue.

The incidence of cardiovascular diseases among women aged 35–54 is rising (37, 38). The unaddressed health needs of fertile women could significantly impact not only live birth rates but also infant health and well-being. In 2015, Yellisinova and coauthors reported that 1,670 per 100,000 children in Kazakhstan were in institutional care, with the country having the highest infant abandonment rate not only among transitional economies of post-Soviet countries but in the world (39). This alarming statistic underscores the importance of targeted healthcare policies. Continuous monitoring and adaptation of healthcare policies are essential to address changing demographic patterns and promote reproductive health (40). Furthermore, Smagulov et al. (41) have also proved a significant association between the crude birth rates in the country and changing climactic, environmental and socio-economic factors.

Strengths of the present analysis: (1) The study utilizes official data from the Statistical Committee of the Republic of Kazakhstan, ensuring reliable and accurate information on live birth rates and the number of women of reproductive age. (2) The use of ITS analysis allows for a detailed examination of both immediate and gradual changes in fertility rates due to the COVID-19 pandemic and the implementation of the CSHIS. (3) By adjusting for the number of women of reproductive age and the effect of CSHIS, the study accounts for significant variables that could influence birth rates, providing a more nuanced understanding of the trends.

Limitations of the study: (1) the study might not account for other factors that could influence fertility rates, such as economic conditions, environmental factors, cultural changes, or other policy interventions. Future studies should consider these additional variables for a more comprehensive analysis. (2) The analysis is based on data up to December 2023. Longer-term data would be beneficial for verifying the sustained impact of the CSHIS on fertility rates. (3) While the study discusses economic instability due to the pandemic, it does not delve deeply into how specific economic policies or changes in employment rates might have influenced fertility decisions. (4) The study assumes that the implementation of CSHIS improved healthcare access and quality uniformly across the population, which may not be the case. Variations in healthcare service delivery and accessibility across different regions and socio-economic groups could also affect birth rates.

## 5 Conclusion

The implementation of the CSHIS, rather than the announcement of the COVID-19 lockdown, has had a significant positive impact on live birth rates in Kazakhstan. When analyzed alone, the pandemic initially led to fluctuations in birth rates, with a notable increase nine months post-lockdown; however, the long-term trends were not sustained, possibly due to economic instability. The CSHIS implementation, aimed at enhancing healthcare access, positively affected fertility rates when considered alongside the COVID-19 lockdown effect and the number of fertile women, highlighting the importance of accessible healthcare services in reproductive decisions. Despite governmental efforts, live birth rates are steadily declining, potentially due to unaddressed health needs of fertile women and economic challenges. Urgent policy-level actions are needed to address gaps in healthcare services and promote reproductive health. Continuous monitoring and adaptation of policies are crucial to mitigate the impact of changing demographic patterns and ensure sustained support for high birth rates in the country.

## Data Availability

Publicly available datasets were analyzed in this study. This data can be found at: https://stat.gov.kz/ru/industries/social-statistics/demography/publications/6351/.

## References

[1] Grundy E Murphy MJ. Demography and public health. 6th ed. Detels R Gulliford M Karim Q Tan C, editors. Oxford, England: Oxford University Press. (2015). Available at: https://eprints.lse.ac.uk/63076/1/__lse.ac.uk_storage_LIBRARY_Secondary_libfile_shared_repository_Content_Grundy,%20E_Demography%20public%20health_Grundy_Demography%20public%20health_2015.pdf (Accessed August 10, 2024).

[2] BBC News. Fertility rate: “Jaw-dropping” global crash in children being born. (2020). Available at: https://www.bbc.com/news/health-53409521 (Accessed August 10, 2024).

[3] Abenova M Myssayev A Kanya L Aldyngurov D. Analysis of maternal and infant health indicators in Kazakhstan: 2003–2018. Open Access Maced J Med Sci. (2021) 9:1133–9. doi: 10.3889/oamjms.2021.7042

[4] United Nations Population Fund (UNFPA). Population Situation Analysis of the Republic of Kazakhstan Summary. (2020) Available at: https://kazakhstan.unfpa.org/sites/default/files/pub-pdf/07_FEB_UNFPA_Report_20pager_ENG_PREVIEW%20%281%29_0.pdf (Accessed August 10, 2024).

[5] Macrotrends. Kazakhstan Birth Rate 1950-2024. (2024) Available at: https://www.macrotrends.net/global-metrics/countries/KAZ/kazakhstan/birth-rate (Accessed August 10, 2024).

[6] Sohrabi C Alsafi Z O’neill N Khan M Kerwan A Al-Jabir A. World Health Organization declares global emergency: A review of the 2019 novel coronavirus (COVID-19). Int J Surg. (2020) 76:71–6. doi: 10.1016/j.ijsu.2020.02.034, PMID: 32112977 PMC7105032

[7] Mallah SI Ghorab OK Al-Salmi S Abdellatif OS Tharmaratnam T Iskandar MA. COVID-19: breaking down a global health crisis. Ann Clin Microbiol Antimicrob. (2021) 20:35–45. doi: 10.1186/s12941-021-00438-7, PMID: 34006330 PMC8129964

[8] Rodela TT Tasnim S Mazumder H Faizah F Sultana A Hossain MM. Economic impacts of coronavirus disease (COVID-19) in developing countries In: Economic Impacts of COVID-19: Working Paper Series (2020).

[9] Phelan N Behan LA Owens L. The impact of the COVID-19 pandemic on women’s reproductive health. Front Endocrinol (Lausanne). (2021) 12:642755. doi: 10.3389/fendo.2021.642755, PMID: 33841334 PMC8030584

[10] Ullah MA Moin AT Araf Y Bhuiyan AR Griffiths MD Gozal D. Potential effects of the COVID-19 pandemic on future birth rate. Front Public Health. (2020) 8:578438. doi: 10.3389/fpubh.2020.578438, PMID: 33363080 PMC7758229

[11] Guetto R Bazzani G Vignoli D. Narratives of the future and fertility decision-making in uncertain times. An application to the COVID-19 pandemic. Vienna Yearb Popul Res. (2022) 20:223–60. doi: 10.1553/populationyearbook2022.res1.6

[12] Kazakhstan-COVID-19 Overview-Johns Hopkins. Available at: https://coronavirus.jhu.edu/region/kazakhstan (Accessed August 5, 2024).

[13] Semenova Y Pivina L Khismetova Z Auyezova A Nurbakyt A Kauysheva A. Anticipating the need for healthcare resources following the escalation of the COVID-19 outbreak in the Republic of Kazakhstan. J Prev Med Public Health. (2020) 53:387–96. doi: 10.3961/jpmph.20.395, PMID: 33296578 PMC7733753

[14] Haruna UA Amos OA Gyeltshen D Colet P Almazan J Ahmadi A. Towards a post-COVID world: Challenges and progress of recovery in Kazakhstan. Public Health Challenges. (2022) 1:e17. doi: 10.1002/puh2.17 PMC12039651 40496378

[15] Sarría-Santamera A Abdukadyrov N Glushkova N Russell Peck D Colet P Yeskendir A. Towards an accurate estimation of COVID-19 cases in Kazakhstan: back-casting and capture–recapture approaches. Medicina (B Aires). (2022) 58:1–9. doi: 10.3390/medicina58020253 PMC8880445 35208577

[16] Semenova Y Kalmatayeva Z Oshibayeva A Mamyrbekova S Kudirbekova A Nurbakyt A. Seropositivity of SARS-CoV-2 in the population of Kazakhstan: A nationwide laboratory-based surveillance. Int J Environ Res Public Health. (2022) 19:2263–77. doi: 10.3390/ijerph19042263, PMID: 35206453 PMC8872132

[17] Nugman A Yegemberdiyeva S Petrovčíková K. Effectiveness of the introduction of compulsory health insurance in the healthcare system of the Republic of Kazakhstan. Viešoji politika ir administravimas. (2022) 21:690–3. doi: 10.13165/VPA-22-21-5-14

[18] World Health Organization. Assessments of sexual, reproductive, maternal, newborn, child and adolescent health in the context of universal health coverage in six countries in the WHO European Region: a synthesis of findings from the country reports. (2020). Available at: https://iris.who.int/handle/10665/331392 (Accessed August 6, 2024).

[19] Almagambetova N. Overhauling the health-care system in Kazakhstan. Lancet. (1999) 354:313–4. doi: 10.1016/s0140-6736(05)75225-9 10440321

[20] Glushkova N Semenova Y Sarria-Santamera A. Public health challenges in post-Soviet countries during and beyond COVID-19. Front Public Health. (2023) 11:1290910–4. doi: 10.3389/fpubh.2023.1290910, PMID: 37886052 PMC10598333

[21] Adilet. On approval of the Concept of Healthcare Development in the Republic of Kazakhstan until 2026. Resolution of the Government of the Republic of Kazakhstan (2022). Available at: https://adilet.zan.kz/rus/docs/P2200000945#z617 (Accessed August 10, 2024).

[22] Mandatory Social Health Insurance (MSHI) | Electronic government of the Republic of Kazakhstan. Available at: https://egov.kz/cms/en/articles/health_care/osms (Accessed August 6, 2024).

[23] Dorjsuren B. Striving to provide universal health coverage in Kazakhstan. Bull World Health Org. (2019) 97:250–1. doi: 10.2471/BLT.19.020419, PMID: 30940980 PMC6438255

[24] Abrokwah SO Moser CM Norton E. The impact of social health insurance on household fertility decisions. J Afr Econ. (2016) 25:699–717. doi: 10.1093/jae/ejw013

[25] Bernal JL Cummins S Gasparrini A. Interrupted time series regression for the evaluation of public health interventions: a tutorial. Int J Epidemiol. (2017) 46:dyw098–355. doi: 10.1093/ije/dyw098, PMID: 27283160 PMC5407170

[26] Bureau of National Statistics Agency for Strategic Planning and Reforms of the Republic of Kazakhstan. Natural population movement of the Republic of Kazakhstan. Demographic Statistics (2023). 1–1. Available at: https://stat.gov.kz/ru/industries/social-statistics/demography/publications/117678/ (Accessed April 25, 2024).

[27] Bureau of National Statistics Agency for Strategic Planning and Reforms of the Republic of Kazakhstan. Population of the Republic of Kazakhstan Demographic Statistics (2023)1–1. Available at: https://stat.gov.kz/ru/industries/social-statistics/demography/publications/157456/ (Accessed April 25, 2024).

[28] Stout MJ Van De Ven CJM Parekh VI Pardo JL Garifullin M Xu M. Use of electronic medical records to estimate changes in pregnancy and birth rates during the COVID-19 pandemic. JAMA Netw Open. (2021) 4:e2111621. doi: 10.1001/jamanetworkopen.2021.11621, PMID: 34081139 PMC8176329

[29] De Rose AF Ambrosini F Mantica G Terrone C. Impact of COVID-19 on birth rate trends in the Italian metropolitan cities of Milan. Genoa Turin Public Health. (2021) 198:35–6. doi: 10.1016/j.puhe.2021.06.026, PMID: 34352613 PMC9451612

[30] Shurenova M Kurakbayev K Abildaev T Tazhieva A. Availability and quality of primary health care in the compulsory health insurance system in Kazakhstan. Medicinski Glasnik (Zenica). (2023) 21:159–65. doi: 10.17392/1675-23 38341755

[31] Abel-Smith B. Health insurance in developing countries: lessons from experience. Health Policy Plan. (1992) 7:215–26. doi: 10.1093/heapol/7.3.215 10121295

[32] Ybrayev Z. COVID-19 in Kazakhstan: Economic consequences and policy implications. COVID-19 Pandemic and Central Asia (2021) 61–66. Available at: https://centralasiaprogram.org/wp-content/uploads/2020/07/CAP_Paper_No.234_by_Zhandos-Ybrayev.pdf (Accessed March 15, 2024).

[33] Barrafrem K Västfjäll D Tinghög G. Financial well-being, COVID-19, and the financial better-than-average-effect. J Behav Exp Finance. (2020) 28:100410–5. doi: 10.1016/j.jbef.2020.100410, PMID: 33042778 PMC7537622

[34] Brodeur A Clark AE Fleche S Powdthavee N. COVID-19, lockdowns and well-being: Evidence from Google Trends. J Public Econ. (2021) 193:104346–54. doi: 10.1016/j.jpubeco.2020.104346, PMID: 33281237 PMC7703221

[35] Usseinova G Turysbekova G Usseinova K Hoffmann T. Maternity Protection in the Republic of Kazakhstan and Abroad: Comparative Legal Analysis. Indian J Sci Technol. (2016) 9:1–9. doi: 10.17485/ijst/2016/v9i27/97680

[36] Suleimenova M Lokshin V Glushkova N Karibayeva S Terzic M. Quality-of-Life Assessment of Women Undergoing In Vitro Fertilization in Kazakhstan. Int J Environ Res Public Health. (2022) 19:13568. doi: 10.3390/ijerph192013568, PMID: 36294148 PMC9603509

[37] Dyusupova A Faizova R Yurkovskaya O Belyaeva T Terekhova T Khismetova A. Clinical characteristics and risk factors for disease severity and mortality of COVID-19 patients with diabetes mellitus in Kazakhstan: a nationwide study. Heliyon. (2021) 7:e06561. doi: 10.1016/j.heliyon.2021.e06561, PMID: 33763618 PMC7972671

[38] Mihajlovic S Nikolic D Milicic B Santric-Milicevic M Glushkova N Nurgalieva Z. Association of pre-pregnancy obesity and COVID-19 with poor pregnancy outcome. J Clin Med. (2023) 12:2936. doi: 10.3390/jcm12082936 37109271 PMC10144693

[39] Yelissinova N Grjibovski AM Yelissinova A Rakhypbekov T Semenova Y Smailova Z. Sociodemographic factors associated with infant abandonment in maternity hospitals in Kazakhstan: A case–control study. Public Health. (2015) 129:1010–3. doi: 10.1016/j.puhe.2015.04.009, PMID: 25986952

[40] Turekulova D Mukhambetova L Beisengaliyev B Orazbayeva K Satkanova R Nurgaliyeva Z. Influence of the Demographic Regions on the Environment: Features and Development Trends. J Environ Manag Tourism. (2021) 12:1796–810. doi: 10.14505/jemt.v12.7(55).06

[41] Smagulov N Zhamantayev O Konkabayeva A Adilbekova A Zhanalina G Shintayeva N. The Role of Climatic, Environmental and Socioeconomic Factors in the Natural Movement of Urban Populations in Kazakhstan, 2012–2020: An Analysis from a Middle-Income Country in Central Asia. Int J Environ Res Public Health. (2024) 21:416–26. doi: 10.3390/ijerph21040416 38673328 PMC11050110

